# Toward a Generalized Hückel Rule: The Electronic Structure of Carbon Nanocones

**DOI:** 10.1021/acs.jpca.1c06402

**Published:** 2021-11-04

**Authors:** Yusuf Bramastya Apriliyanto, Stefano Battaglia, Stefano Evangelisti, Noelia Faginas-Lago, Thierry Leininger, Andrea Lombardi

**Affiliations:** † Laboratoire de Chimie et Physique Quantiques-IRSAMC, Université de Toulouse et CNRS, 118, Route de Narbonne, F-31062 Toulouse Cedex, France; ‡ Dipartimento di Chimica, Biologia e Biotecnologie, Università degli Studi di Perugia, Via Elce di Sotto 8, I-06123 Perugia, Italy; § Department of Chemistry-BMC, Uppsala University, P.O. Box 576, SE-75123 Uppsala, Sweden

## Abstract

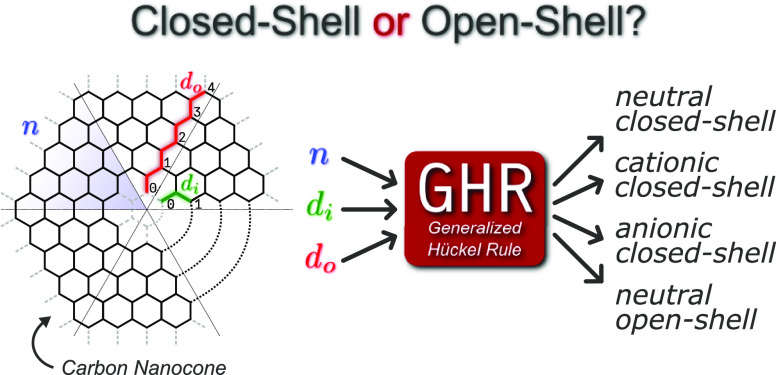

In this work, we investigate a particular class of carbon nanocones, which we name graphannulenes, and present a generalized Hückel rule (GHR) that predicts the character of their ground state based on simply the three topological indices that uniquely define them. Importantly, this rule applies to both flat and curved systems, encompassing a wide variety of known structures that do not satisfy the “classic” 4*n* + 2 rule such as coronene, corannulene, and Kekulene. We test this rule at the Hückel level of theory for a large number of systems, including structures that are convex and flat, with a saddle-like geometry, and at the CASSCF level of theory for a selected representative subset. All the performed calculations support the GHR that we propose in this work.

## Introduction

The extraordinary ability of carbon to concatenate with other carbon atoms, thus forming large stable edifices possibly containing other types of atoms, is at the basis of the unique behavior of this eclectic element, and ultimately of life itself. Besides the standard three-dimensional allotropes, carbon is able to form structures that are essentially zero dimensional (such as fullerenes), one dimensional (polyenes, nanotubes), and two dimensional (graphene). Among all these possible edifices, a particular class of systems that has received less attention than others is that of carbon nanocones, whose first successful synthesis was achieved shortly after that of carbon nanotubes.^1−4^ In contrast to the latter, however, the synthesis of nanocones has two advantages: no catalytic metal is in general involved and they can be mass-produced at room temperature.^5^ This, combined with their many interesting properties, makes carbon nanocones a scientifically and technologically relevant class of nanostructures. In the past two decades, a notable amount of effort has been put into the structural characterization, both electronic and geometrical, of these systems,^6−16^ and they have been proposed for a number of applications^5^ encompassing gas storage,^14,17^ functionalization,^18−20^ electronic applications,^21^ and as optical materials.^22^ In the literature, two related names are used for these systems: carbon nanocones and carbon nanohorns.^5,12,23^ However, their definition starts from the same rationale: triangular graphene fragments are merged together, with the tip of the nanocone (or nanohorn) defining the curvature and the particular topological nature of the molecular system. In this work, we focus on a particular type of nanocones, those that have a single annulene ring at their tip. These graphene-derived structures, obtained by growing a triangular sector of graphene surface by starting from each carbon atom belonging to an annulene ring, form a very general class of systems that encompass many well-known polycyclic aromatic hydrocarbons (PAHs) such as benzene (C_6_H_6_), corannulene (C_20_H_10_), coronene (C_24_H_12_), and Kekulene (C_48_H_24_). Because cyclic polyenes are named annulenes, we will use the term *graphannulenes* (cfr. also corannulene) to indicate the resulting structures, noting in passing that the former can be seen as a special case of the latter. A common theme of discussion for these systems is their stability, which is often related to the concept of aromaticity and the electronic structure properties of their ground state.^24−31^ While many descriptors for aromaticity have been developed in the last decades (see *e.g.,* Solà^32^ and references therein), they usually rely on high-level calculations, for example, to obtain nucleus-independent chemical shift values^33^ or the plots of anisotropy of the induced current density.^34^ A simple and intuitive rule, such as the Hückel rule for annulenes, is still missing for more complicated structures, for instance nanocones, which is probably one of the reasons why this rule is sometimes (mis)used for more general PAHs. In its original formulation, Hückel’s “4*n* + 2 rule for aromaticity”^35,36^ states that planar, monocyclic conjugated polyenes, that is, annulenes, are aromatic if the total number of electrons in the π system is equal to 4*n* + 2, where *n* is a positive integer. On the other hand, if it is equal to 4*n*, then, the annulene is said to be antiaromatic.^37^ In this context, (anti)aromaticity is understood in terms of molecular (in)stability with respect to the open-chain counterpart. The increased stability of aromatic rings with respect to open chains can be related to the width of the HOMO–LUMO gap that in particular, for the case of small rings, is much larger with respect to the corresponding open systems. This means that the annulenes with 4*n* + 2 electrons are energetically more stable than linear polyenes, while the opposite is true for systems with 4*n* electrons. From an electronic structure perspective, the increased stability of the 4*n* + 2 cyclic system is a manifestation of the closed-shell character of the ground state wave function. In this case, according to Hückel’s molecular orbital (HMO) theory, all π bonding orbitals are doubly occupied, whereas all antibonding ones are empty, resulting in a large HOMO–LUMO gap. Conversely, the decreased stability of 4*n* rings with respect to the chain conformation is due to the presence of two degenerate non-bonding orbitals at the Fermi level, which are only partially occupied by two electrons. This results in an unstable open-shell singlet wave function that in practice, favors bond-length alternation as observed in the prototypical example of the cyclobutadiene (CBD) molecule.^38,39^ Note that when dealing with a more complex structure than polyene systems, the notion of aromaticity becomes more complex as well and is not anymore a synonym of stability as it is in the context of the original Hückel rule. This is for instance explored in a recent work by Zdetsis,^40^ where an intimate connection between aromaticity, HOMO–LUMO gap, and π electron counts is established for several types of PAHs.

The original Hückel rule considers the number of π electrons as the basic quantity to determine the stability of an annulene. However, when dealing with the larger class of systems represented by graphannulenes, it is useful to restate it in a slightly different, but equivalent manner. That is, instead of considering the number of π electrons as the basic quantity, we use the number *N* of sp^2^ carbon atoms. For example, benzene, cyclodecatetraene, and so forth have *N* = 4*n* + 2 carbon atoms, while CBD, cyclooctatetraene, and so forth have *N* = 4*n* carbon atoms. These systems have the same number of sp^2^ carbon atoms as π electrons, and thus, there is a one-to-one correspondence between them. It is then conceptually easy to extend this simple rule to odd-atom rings, whereby *N* = 4*n* + 3 annulenes can be shown to have a *cationic closed-shell* ground state, while *N* = 4*n* + 1 structures are characterized by an *anionic closed-shell* wave function. This simply boils down to either adding or removing one π electron from the annulene such that it has a total of 4*n* + 2. What is far less trivial is instead the extension to the more general class of systems composed of an arbitrary number of concentric annulenes bonded together. To the best of our knowledge, the only attempt in this direction to date is found in the work by Zdetsis,^41^ whereby a novel rule for concentric hexagonal PAHs is derived in analogy to the atomic shell structure. In the present article instead, we focus our attention to a more general class of systems and in the spirit of Hückel’s early works, we shall present a generalized rule rooted in HMO theory that applies to *all* graphannulenes, as much as the original Hückel rule applies to *all* annulenes. This new rule is a generalization of the original one, which is nevertheless contained as a special case, and thus we shall call it the generalized Hückel rule (GHR). The GHR is tested both at the Hückel level of theory and with multiconfigurational *ab initio* methods, providing evidence to support its validity.

The article is organized as follows: in the section Graphannulenes, we introduce the particular class of systems that are subject of this work; in the following section, we present the GHR; the third section is dedicated to the *ab initio* calculations, and in the last Conclusions section, we summarize our work and discuss future directions.

## Graphannulenes

**Figure 1:**
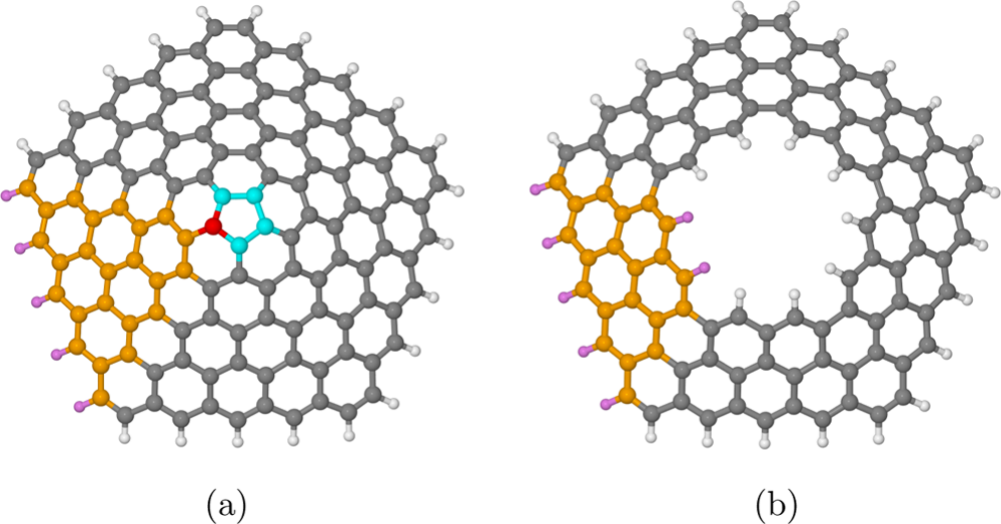
(a) Conical hat structure for *n* = 5 built by growing each triangular sector starting from the central carbon ring. The latter is color-coded in cyan, a single graphene triangle is highlighted in orange, with the corresponding connecting carbon atom of the central ring in red. Purple is used for the saturating hydrogen atoms. (b) Topological annulus, which, roughly speaking, corresponds to a truncated cone, for *n* = 5 built by deleting the two innermost rings of the complete cone structure.

The structures considered in the present work have highly symmetric geometries.^13,15^ Graphene nanocones of order *n* can be obtained by ideally cutting *n* identical equilateral triangular portions out of a graphene surface, and merging the resulting fragments in a suitable way.^6,10,14^ The process can be started from a carbon ring containing *n* atoms, to which the *n* identical triangular graphene sheets are connected: one of the three apical carbon atoms of each of the *n* graphene sheets is bounded to a corresponding carbon atom belonging to the central chain, the neighboring sheet edges are connected by pairs and the remaining open edges are saturated by hydrogen atoms. In this way, a conical structure is obtained, as shown in Figure 1a for the case *n* = 5. Broadly speaking, the resulting surface will be a cone having a positive curvature for *n* < 6, a saddle-like structure having negative curvature for *n* > 6, while in the particular case *n* = 6, one obtains a flat hexagonal graphene nanoisland. For this reason, the graphannulene family is much larger than the simple graphene nanocones.

**Figure 2:**
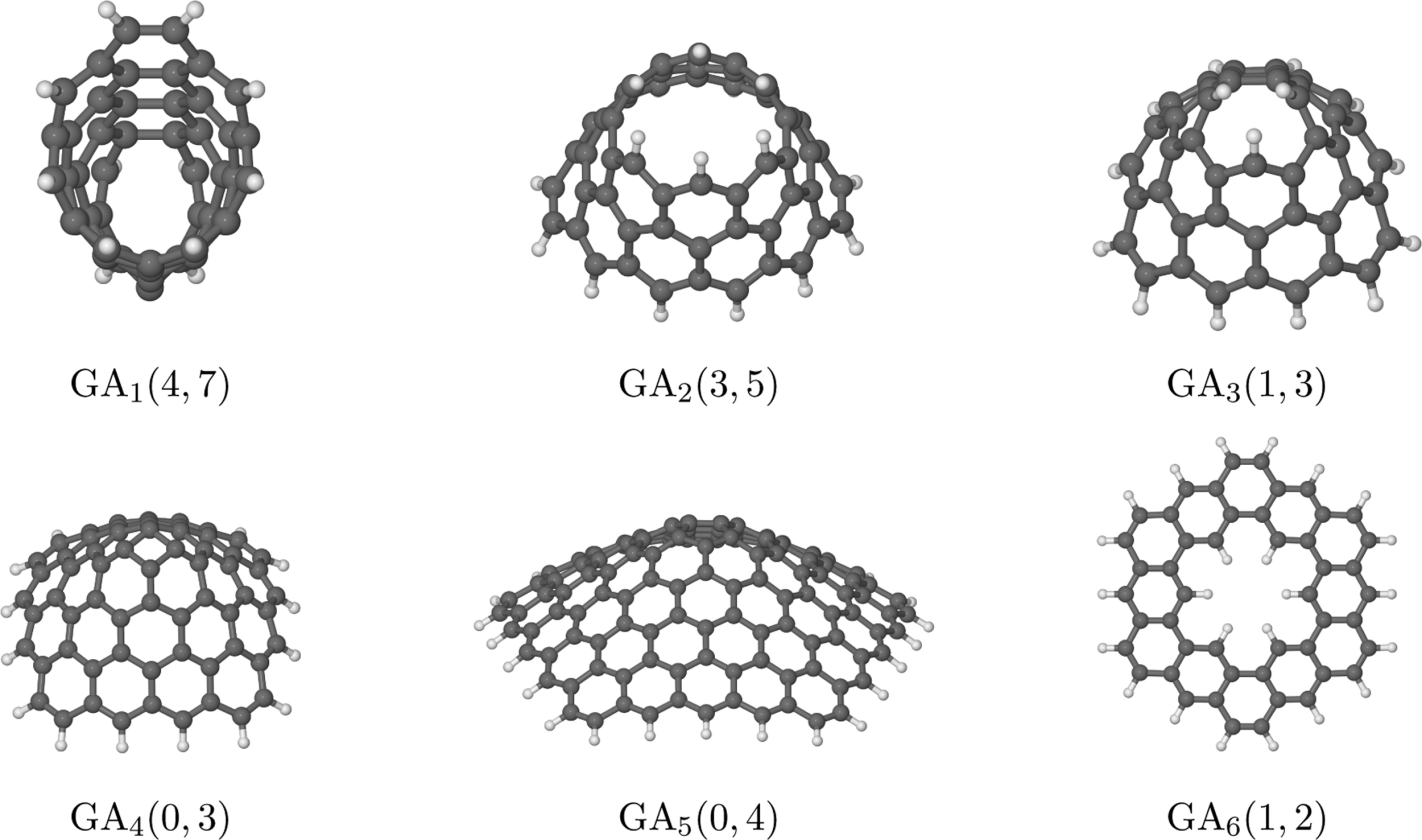
Some examples of graphannulenes following the nomenclature GA_*n*_(*d*_i_, *d*_o_) introduced in the text for both complete and truncated structures with *n* ≤ 6.

These structures can also be seen as the result of placing a series of concentric closed carbon chains around a central ring (a closed carbon chain of *n* atoms or equivalently speaking an [*n*]annulene without hydrogens). From a purely topological point of view, disregarding the exact three-dimensional shape, their structure is isomorphic to a disk. If, at the end of the process, some of the innermost rings are deleted, a *topological annulus* is obtained as illustrated in Figure 1b, again for the case *n* = 5. In all cases, at the end of the whole construction process, all carbons having only two bonds are saturated with a hydrogen atom, thus leaving all the carbons in a sp^2^ hybridization state. For this reason, each carbon atom gives exactly one electron to the resulting π system. Notice that, if the central ring is present, one has a *complete cone*, and there is only *one* outer edge bearing the H atoms (Figure 1a). In the case of a *truncated cone* (also called a *conical frustrum*), on the other hand, *two* edges (an inner and an outer one) that bear H atoms are present (Figure 1b). Because of its regularity and high symmetry, the topology of a regular graphannulene is completely defined by the order *n* of the central ring, and the two “topological distances” of the innermost and outermost carbon rings, *d*_i_ and *d*_o_, respectively, from the central one. The central ring can be either present or absent; the distance *d* is zero for the carbon atoms belonging to this ring, one for those belonging to the ring immediately surrounding it, two for the next one, and so on. The resulting graphannulene structure will be indicated as GA_*n*_(*d*_i_, *d*_o_), with *n* ≥ 1 and 0 ≤ *d*_i_ ≤ *d*_o_. If only the central ring is present, both topological distances will be zero, and we will have the GA_*n*_(0, 0) system. In general, *d*_i_ represents the number of inner rings that is deleted in the building process, so the number of concentric rings is given by the difference *d*_o_ – *d*_i_ + 1. By adopting this notation, an annulene ring containing, for instance, *n* carbon atoms will be indicated as GA_*n*_(0, 0): benzene will become GA_6_(0, 0), cyclopentadienyl radical will be GA_5_(0, 0), and so on. In the same way, coronene will be indicated by GA_6_(0, 1), corannulene by GA_5_(0, 1), Kekulene by GA_6_(1, 2), and the structures presented in Figure 1a,b by GA_5_(0, 4) and GA_5_(2, 4), respectively. For *n* = 6, and if no inner rings are deleted, one gets the hexagonal flat structures of the coronene family (which were the central topic of the work by Zdetsis^41^), having *D*_6*h*_ symmetry, and in the limit case of infinite triangles, the complete graphene surface. On the other hand, for 0 < *n* < 6, the resulting structures are no-longer flat and have a *C*_*nv*_ symmetry (note that the *C*_1*v*_ group is actually indicated as *C*_*s*_, and no symmetry axis is present). Finally, if *n* > 6, the resulting surfaces have a saddle-like shape, and the symmetry pattern can be more complicated. Given the large amount of possible structures with *n* > 6, in this work, we focus our attention mainly on the case *n* ≤ 6, leaving the *n* > 6 possibility for future investigations. Some examples of these structures are illustrated in Figure 2.

Notice that the smallest annulene ring is a three-carbon chain. Therefore, in principle, *n* should be an integer number greater than 2, while *d*_i_ and *d*_o_ are non-negative integers, with *d*_i_ ≤ *d*_o_. However, when the central ring is missing, *n* can also take the values of 1 or 2, as discussed later.

The symmetry of the graphannulenes plays a very important role in their wave function structure, both at tight-binding and *ab initio* level of theory. Let us consider the Hückel Hamiltonian symmetry first. From a topological point of view, and in absence of dimerization, the GA_*n*_(0, *d*_o_) structures are isomorphic to a regular polygon having *n* sides, and their space symmetry group is therefore *D*_*nh*_. However, because all the atomic Hückel orbitals of the system have a *p* character, they all behave in the same way (anti-symmetrically) under reflection with respect to the σ_h_ plane. This means that the subgroup *C*_*nv*_ of the whole *D*_*nh*_ group is enough to describe the symmetry properties of the MOs. At *ab initio* level of theory, on the other hand, one must consider the real spatial disposition of the atoms. The system space symmetry will depend in a crucial way on the order *n* of the principal symmetry axis, *C*_*n*_. If *n* is smaller than six, the structures will have conical shapes, and a symmetry group *C*_*nv*_. For *n* = 6, on the other hand, flat edifices are obtained, with symmetry *D*_6*h*_. The situation is much more complex when *n* > 6. Preliminary results indicate a *D*_2*d*_ symmetry if *n* is an integer multiple of four, *C*_2*v*_ if *n* is a multiple of two but not of four, and *C*_*s*_ if *n* is odd. However, different symmetry groups are also possible, but they will not be taken into account here. In the present contribution, we consider any value of *n* for the Hückel Hamiltonian, but we limit our *ab initio* investigations to the cases 1 ≤ *n* ≤ 6.

### Generalized Hückel Rule

Due to the topological nature of the Hückel Hamiltonian, it is possible to perform *tight-binding* calculations on systems whose atom disposition is actually impossible in the real 3D space. The ground-state nature of these systems depends in a crucial way on the order *n* of the symmetry rotation, which can be arbitrarily large at *tight-binding* level, and the difference between the inner and outer ring numbers. The *tight-binding* energy spectrum of a graphannulene is strictly related to the one of graphene. In fact, a GA_*n*_(*d*_i_, *d*_o_) structure is composed of *n* graphene fragments, whose size is, proportional to the difference *d*_o_ – *d*_i_. For this reason, it is not surprising that the spectrum of the system converges, for any *fixed* value of *n* and in the limit *d*_o_ – *d*_i_ → ∞, to the energy spectrum of the infinite graphene sheet. For small systems, on the other hand, there are finite-size effects that modify the spectrum in a significant way. Notice that, in the opposite limit, *d*_o_ – *d*_i_ = 0, one obtains an annulene ring, the *C*_*n*(2*d*_i_+1)_ system.

In the case of finite-size systems, the *tight-binding* energy spectrum, and hence, the ground-state wave function, strongly depend on the total number of electrons. To discuss its structure, it is useful to recall the well-known Hückel Rule^35,36^ that predicts the nature of the ground-state wave function for annulenes. Let *l* be the number of carbon atoms of the chain. If *l* is even, it can be either of the form *l* = 4*m* or *l* = 4*m* + 2, where *m* is a non-negative integer number. The Hückel rule says that annulenes containing *l* = 4*m* + 2 carbon atoms have a closed-shell wave function because the Fermi level (the HOMO–LUMO frontier) passes between a pair of filled and one of empty degenerate orbitals. Cycles containing *l* = 4*m* carbon atoms, on the other hand, are characterized by a pair of degenerate half-filled orbitals at the Fermi level. Because these two orbitals host only two electrons, the wave function has an open-shell character. If *l* is odd, neutral cycles have an odd number of π electrons, and the wave function is characterized by at least one unpaired electron. In this case, the cycles of length *l* = 4*m* + 1 have the uppermost occupied pair of degenerate orbitals hosting three electrons. This means that these orbitals can host an additional electron, and the anionic species is a closed-shell (indicated as CS^–^). This is the well-known case of the cyclopentadienyl anion, the most common anion in organic chemistry. Cycles of the length *l* = 4*m* + 3, on the other hand, have the uppermost occupied pair of degenerate orbitals hosting only one electron. In this case, it is the cationic species that is a closed-shell, denoted as CS^+^.

In order to summarize this behavior, we define the *integer typep* of the integer number *l* according to the formula *l* = 4*m* + *p*, with *p* = 0, 1, 2, 3. We will use the notation  in order to indicate the integer type of *l*. Using this notation, the Hückel rules for annulenes can be summarized as follows:

(1) 

(2) 

(3) 

(4) 

In other words, the nature of the ground state in an annulene depends in a unique way on the integer type of the annulene length *l*.

In a very similar way, the nature of the wave function in graphannulenes depends on the values of three quantities: the order *n* of the graphannulene, and the order *d*_i_ and *d*_o_ of the innermost and outermost carbon rings, respectively. Notice that, if *d*_o_ – *d*_i_ is even, *d*_o_ and *d*_i_ are both even or both odd. The situation is synthesized in the next scheme:

(1) If the number of rings is even (hence, *d*_o_ – *d*_i_ is odd), the system is always CS.
(2) If the number of rings is odd (hence, *d*_o_ – *d*_i_ is even), then:
  (a)
  (b) then:
    (i) *d*_i_ and *d*_o_ even ⇒ CS^–^
    (ii) *d*_i_ and *d*_o_ odd ⇒ CS^+^
  (c)
  (d) then:
    (i) *d*_i_ and *d*_o_ even ⇒ CS^+^
    (ii) *d*_i_ and *d*_o_ odd ⇒ CS^–^

We do not have a formal proof of this rule yet, but it has been numerically verified for all the radii 0 ≤ *d*_i_ ≤ *d*_o_ ≤ 6 up to very large values of *n* (see Supporting Information for details regarding these calculations). Work is in progress for a general demonstration. Cyclic polyenes have one ring around the system symmetry axis, and *d*_i_ = *d*_o_ = 0 are both even. We are in case 2, and one can verify that the usual Hückel rule is easily recovered. On the other hand, coronene, corannulene, Kekulene, and similar systems having *one* crown of benzene rings around an inner annulene are characterized by two carbon rings around the symmetry axis. Therefore, we are in case 1, and these molecules are predicted to be all closed-shells, as indeed it is the case.^24,25,28,42^ This, despite the fact that they have 20, 24 and 48 π electrons, respectively, which would seem apparently to be in contradiction with the Hückel rule. Furthermore, the GHR does not distinguish between flat and non-flat systems because it is based on topological features rather than geometrical ones; as such it applies to systems such as the bowl-shaped corannulene molecule or other conical structures (see *e.g.,*Figure 2). Nevertheless, not all curved systems are encompassed by the GHR: a notable example is that of fullerenes, which have a different type of curvature than graphannulenes and are therefore completely different objects. In this context, the GHR is complementary to the more famous Hirsch rule^43^ and generalization thereof.^44^ This GHR also predicts correctly the closed-shell character in relation to the aromaticity of circumcoronene (GA_6_(0, 2)).^26,33,45^ It is important to stress out that the GHR predicts the nature of the ground state based on topological features of the graphannulenes structure, whereby a closed-shell wave function usually implies a stable molecule. Hence, no information can be obtained on the particular global or local aromatic character of the system. An illustrative example is that of coronene, whose stability is well-established and correctly predicted by the GHR, but its aromaticity is subject to different interpretations.^46,47^ Furthermore, because no quantitative measure can be extracted from the rule, no relative stability comparison between systems with the same ground state character is possible, but in general only between the open-shell and the closed-shell ones.

### *Ab Initio* Calculations

To corroborate the results obtained with the Hückel Hamiltonian, we carried out *ab initio* multiconfigurational calculations on a number of nanocones encompassing all possible GHR outcomes for the values *n* ≤ 6 and combinations of *d*_i_ and *d*_o_. In particular, we have performed state-average complete active space self-consistent field (SA-CASSCF)^48,49^ calculations of 13 illustrative systems, whereby the lowest singlet and triplet states were targeted during the optimization, so that the spin symmetry and the nature of the ground state could be analyzed.

## Computational Details

The systems considered in this section are listed in the first column of Table 1. All geometries were optimized with restricted Kohn–Sham density functional theory (DFT) using the 6-31G* basis set^50^ and the B3LYP exchange and correlation functional^51−53^ in Gaussian 16, version B01.^54^ For all structures, we optimized the closed-shell singlet state of either the neutral, cationic, or anionic system according to the prediction of the GHR, and checked that it constituted a true minimum of the potential energy surface through a frequency calculation. Inclusion of diffuse functions, that is, using the basis set 6-31+G*, for an illustrative anionic system, GA_5_(0, 2), did not result in any significant difference in the structure. In the case of a predicted open-shell character, we first optimized the geometry of the lowest triplet state using unrestricted DFT and then used this structure to optimize the geometry of the broken symmetry singlet. In this case, the frequency calculation expectedly yielded one imaginary frequency as the symmetric conformation on the singlet surface is a transition state between the two dimerized minima, similar to CBD. The imaginary mode breaks the symmetry according to a Jahn–Teller distortion. All optimized structures belong to their highest molecular point group, that is *C*_*nv*_, and are available as xyz files as part of the Supporting Information.

**Table 1:** SA-CASSCF Computational Details and Results for a Series of Illustrative Graphannulenes^a^

system	# C atoms	GHR	AS	*S* = 0	*S* = 1	*ab initio*	ref. weight
GA_1_(5, 7)	39	CS^+^	(8,8)	1	1	CS^+^	0.87342
GA_1_(6, 8)	45	CS^–^	(8,8)	1	1	CS^–^	0.84241
GA_1_(7, 8)	32	CS	(8,8)	1	1	CS	0.77827
GA_2_(3, 4)	32	CS	(8,8)	1	1	CS	0.86966
GA_3_(0, 2)	27	CS^+^	(8,8)	1	2	CS^+^	0.91387
GA_3_(1, 3)	45	CS^–^	(8,8)	1	2	CS^–^	0.84350
GA_3_(2, 3)	36	CS	(8,8)	1	2	CS	0.92921
GA_4_(0, 2)	36	OS	(12,12)	1	1	OS	0.39/0.39
GA_4_(1, 2)	32	CS	(12,12)	1	4	CS	0.88186
GA_5_(0, 1)	20	CS	(8,8)	1	4	CS	0.92900
GA_5_(0, 2)	45	CS^–^	(8,8)	1	4	CS^–^	0.94635
GA_5_(1, 3)	75	CS^+^	(8,8)	1	4	CS^+^	0.93588
GA_6_(0, 1)	24	CS	(8,8)	1	4	CS	0.93148

a # C atoms shows the number of carbon atoms in the corresponding system, GHR is the prediction of the generalized Hückel rule, AS shows the active space used in the calculation, *S* = 0 and *S* = 1 indicate the number of states with singlet and triplet spin symmetry, respectively, included in the state-average optimization, *ab initio* indicates the wave function character obtained with the SA-CASSCF calculation and ref. weight is the largest weight (coefficient squared) of the ground state wave function.

Subsequently, in order to assess the electronic structure of the ground state wave function and thus either confirm or refute the prediction of the GHR, we carried out state-average CASSCF single point calculations on all systems that are considered. The basis set used in this case was the def2-SV(P) basis set,^55^ together with the density fitting (RIJK) approximation and the auxiliary def2/JK universal basis set.^56^ The active spaces and the number of states included in the calculations were selected according to the orbital energy and degeneracy obtained from the Hartree–Fock calculations and can be consulted in Table 1. No point group symmetry was enforced in this part. All SA-CASSCF calculations were carried with the ORCA program package, version 4.2.1.^57^

## Results

The most important details of the systems considered and the results obtained are summarized in Table 1.

In all cases, we have found that the ground state wave function was a singlet, and its composition reflected the character predicted by the GHR, with no exception. In particular, all systems predicted with a closed-shell ground state (CS, CS^+^ or CS^–^) are dominated by a single configuration with a corresponding weight of at least 0.77. Hence, these systems are essentially single reference. On the other hand, the ground state wave function of the system predicted to be open-shell, GA_4_(0, 2), is dominated by two degenerate configurations with a weight of 0.39 each. This case is analogous to CBD, whereby the symmetric system is actually a transition state on the ground state potential energy surface between two dimerized minima. Nevertheless, as for CBD in the framework of the original Hückel rule, the open-shell character predicted by the GHR for GA_4_(0, 2) is a signature of the instability of the symmetric conformation. Note that this open-shell character of the *ground state* is only true within HMO theory, whereby the frontier orbitals are exactly degenerate by construction. In reality, full relaxation of GA_4_(0, 2) results in a Jahn–Teller distortion and a ground state which is closed-shell similarly to CBD.

**Figure 3:**
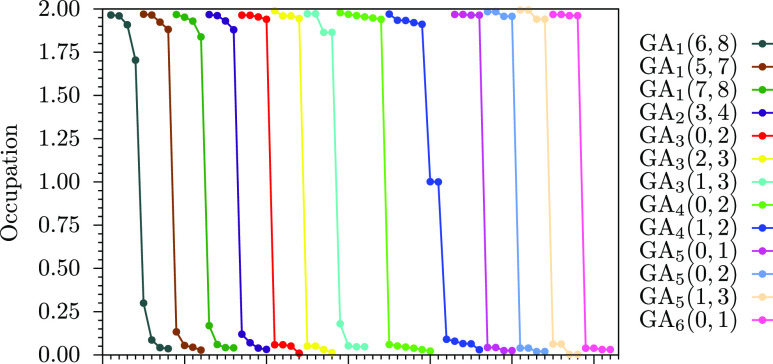
NOONs of the graphannulenes studied at the CASSCF level of theory. Each curve on this plot represents a graphannulene, and the circles within the a graphannulene correspond to the occupation number of the active orbitals in the ground state wave function. The *x*-axis simply indexes the orbitals. The curves are plotted such that they do not overlap and the NOONs for all graphannulenes are clearly visible.

Further evidence on the wave function character is provided by the natural orbital occupation numbers (NOONs); these are trivially 2 and 0 for the occupied and virtual orbitals, respectively, and any number between 0 and 2 for the active orbitals. In Figure 3, we plot the NOONs of the ground state CASSCF wave function for all systems considered at the *ab initio* level and, as can be seen, for all closed-shell systems, these are largely close to either 2 or 0. On the other hand, the GA_4_(0, 2) graphannulene has two orbitals with an occupation number of approximately 1, highlighting the open-shell character of its ground state and the presence of two unpaired electrons. This is in agreement with the GHR and the observations from Table 1.

## Conclusions

To summarize, in this work, we propose a generalization of the Hückel rule, that is able to predict the nature of the ground state wave function for graphannulene systems solely based on the three topological indices that uniquely define them. At the Hückel level of theory, the GHR was verified for all values of 0 ≤ *d*_i_ ≤ *d*_o_ ≤ 6, and within this range, tested for a large number of *n* values. At the moment, we are working on an analytical general proof of the rule that is valid for arbitrary values of the three topological indices. Corroborating the semi-empirical approach, SA-CASSCF calculations on 13 systems with *n* ≤ 6 also resulted in ground state wave functions perfectly matching the GHR prediction, based on an analysis of the wave function coefficients and NOONs. Importantly, while we did not focus our attention on the connection between the GHR and the many existing aromaticity descriptors (which we leave for future dedicated investigations), the nature of the wave function results in a good indicator for the stability of the system, in complete analogy to the more famous 4*n* + 2 Hückel rule. Indeed, the GHR is an effective generalization of the latter, which, however, is applicable to conjugated π systems that no longer need to be planar and is formally derived to comprise systems beyond the simple annulene series. Notably, this rule correctly predicts the wave function character of many systems that are stable, considered aromatic and that *do not* satisfy the 4*n* + 2 rule such as coronene, corannulene and Kekulene. We believe that the GHR constitutes an important tool for the organic chemist, allowing to quickly infer the stability of a large number of PAHs without relying on expensive calculations. At last, we should note that while the prediction of the GHR for the systems considered in this work was verified empirically, a full analytical demonstration of the rule is far from being trivial, and at the moment, work is in progress in this direction.

## Supporting Information Available

All geometries used in this work (ZIP)
Details of the tight-binding calculations (PDF)

## References

[ref1] Ge M.; Sattler K. Observation of fullerene cones. Chem. Phys. Lett. 1994, 220, 192–196. 10.1016/0009-2614(94)00167-7.

[ref2] Endo M.; Takeuchi K.; Kobori K.; Takahashi K.; Kroto H. W.; Sarkar A. Pyrolytic carbon nanotubes from vapor-grown carbon fibers. Carbon 1995, 33, 873–881. 10.1016/0008-6223(95)00016-7.

[ref3] Krishnan A.; Dujardin E.; Treacy M. M. J.; Hugdahl J.; Lynum S.; Ebbesen T. W. Graphitic cones and the nucleation of curved carbon surfaces. Nature 1997, 388, 451–454. 10.1038/41284.

[ref4] Iijima S.; Yudasaka M.; Yamada R.; Bandow S.; Suenaga K.; Kokai F.; Takahashi K. Nano-aggregates of single-walled graphitic carbon nano-horns. Chem. Phys. Lett. 1999, 309, 165–170. 10.1016/s0009-2614(99)00642-9.

[ref5] Karousis N.; Suarez-Martinez I.; Ewels C. P.; Tagmatarchis N. Structure, Properties, Functionalization, and Applications of Carbon Nanohorns. Chem. Rev. 2016, 116, 4850–4883. 10.1021/acs.chemrev.5b00611. 27074223

[ref6] Charlier J.-C.; Rignanese G.-M. Electronic Structure of Carbon Nanocones. Phys. Rev. Lett. 2001, 86, 5970–5973. 10.1103/physrevlett.86.5970. 11415406

[ref7] Klein D. J. Topo-combinatoric categorization of quasi-local graphitic defects. Phys. Chem. Chem. Phys. 2002, 4, 2099–2110. 10.1039/b110618j.

[ref8] Mota R.; Machado M.; Piquini P. Structural and Electronic Properties of 240°Nanocones. Phys. Status Solidi A 2003, 0, 799–802. 10.1002/pssc.200306216.

[ref9] Terrones H.; Terrones M. Curved nanostructured materials. New J. Phys. 2003, 5, 126 10.1088/1367-2630/5/1/126.

[ref10] Jordan S. P.; Crespi V. H. Theory of Carbon Nanocones: Mechanical Chiral Inversion of a Micron-Scale Three-Dimensional Object. Phys. Rev. Lett. 2004, 93, 255504 10.1103/physrevlett.93.255504. 15697907

[ref11] Muñoz-Navia M.; Dorantes-Dávila J.; Terrones M.; Terrones H. Ground-state electronic structure of nanoscale carbon cones. Phys. Rev. B: Condens. Matter Mater. Phys. 2005, 72, 235403 10.1103/physrevb.72.235403.

[ref12] Klein D. J.; Balaban A. T. The Eight Classes of Positive-Curvature Graphitic Nanocones. J. Chem. Inf. Model. 2006, 46, 307–320. 10.1021/ci0503356. 16426066

[ref13] Lin C.-T.; Lee C.-Y.; Chiu H.-T.; Chin T.-S. Graphene Structure in Carbon Nanocones and Nanodiscs. Langmuir 2007, 23, 12806–12810. 10.1021/la701949k. 18001066

[ref14] Heiberg-Andersen H.; Skjeltorp A. T.; Sattler K. Carbon nanocones: A variety of non-crystalline graphite. J. Non-Cryst. Solids 2008, 354, 5247–5249. 10.1016/j.jnoncrysol.2008.06.120.

[ref15] Naess S. N.; Elgsaeter A.; Helgesen G.; Knudsen K. D. Carbon nanocones: wall structure and morphology. Sci. Technol. Adv. Mater. 2009, 10, 065002 10.1088/1468-6996/10/6/065002. 27877312 PMC5074450

[ref16] Cox B.; Hill J. Carbon Nanocones with Curvature Effects Close to the Vertex. Nanomaterials 2018, 8, 624 10.3390/nano8080624. PMC6116322 30126125

[ref17] Adisa O. O.; Cox B. J.; Hill J. M. Open Carbon Nanocones as Candidates for Gas Storage. J. Phys. Chem. C 2011, 115, 24528–24533. 10.1021/jp2069094.

[ref18] Pagona G.; Tagmatarchis N.; Fan J.; Yudasaka M.; Iijima S. Cone-End Functionalization of Carbon Nanohorns. Chem. Mater. 2006, 18, 3918–3920. 10.1021/cm0604864.

[ref19] Trzaskowski B.; Jalbout A. F.; Adamowicz L. Functionalization of carbon nanocones by free radicals: A theoretical study. Chem. Phys. Lett. 2007, 444, 314–318. 10.1016/j.cplett.2007.07.045.

[ref20] Suarez-Martinez I.; Mittal J.; Allouche H.; Pacheco M.; Monthioux M.; Razafinimanana M.; Ewels C. P. Fullerene attachment to sharp-angle nanocones mediated by covalent oxygen bridging. Carbon 2013, 54, 149–154. 10.1016/j.carbon.2012.11.014.

[ref21] Lu X.; Yang Q.; Xiao C.; Hirose A. Field electron emission of carbon-based nanocone films. Appl. Phys. A 2006, 82, 293–296. 10.1007/s00339-005-3410-2.

[ref22] Hu Y.-Y.; Sun S.-L.; Zhong R.-L.; Xu H.-L.; Su Z.-M. Novel Trumpet-Shaped Conjugation Bridge (Carbon Nanocone) for Nonlinear Optical Materials. J. Phys. Chem. C 2011, 115, 18545–18551. 10.1021/jp2069336.

[ref23] Suarez-Martinez I.; Grobert N.; Ewels C. P. Nomenclature of sp2 carbon nanoforms. Carbon 2012, 50, 741–747. 10.1016/j.carbon.2011.11.002.

[ref24] Cyvin S. J.; Brendsdal E.; Brunvoll J.; Skaret M. Corannulene as a member of circulenes: its topological properties and molecular vibrations. J. Mol. Struct. 1991, 247, 119–127. 10.1016/0022-2860(91)87068-s.

[ref25] Jiao H.; Schleyer P. v. R. Is Kekulene Really Superaromatic?. Angew. Chem., Int. Ed. 1996, 35, 2383–2386. 10.1002/anie.199623831.

[ref26] Soncini A.; Steiner E.; Fowler P. W.; Havenith R. W. A.; Jenneskens L. W. Perimeter Effects on Ring Currents in Polycyclic Aromatic Hydrocarbons: Circumcoronene and Two Hexabenzocoronenes. Chem.—Eur. J. 2003, 9, 2974–2981. 10.1002/chem.200204183.

[ref27] Balaban A. T.; Klein D. J. Claromatic Carbon Nanostructures. J. Phys. Chem. C 2009, 113, 19123–19133. 10.1021/jp9082618.

[ref28] Dobrowolski M. A.; Ciesielski A.; Cyrański M. K. On the aromatic stabilization of corannulene and coronene. Phys. Chem. Chem. Phys. 2011, 13, 20557 10.1039/c1cp21994d. 21922090

[ref29] Fowler P. W.; Soncini A. Visualising aromaticity of bowl-shaped molecules. Phys. Chem. Chem. Phys. 2011, 13, 20637 10.1039/c1cp21931f. 21922089

[ref30] Popov I. A.; Boldyrev A. I. Chemical Bonding in Coronene, Isocoronene, and Circumcoronene. Eur. J. Org. Chem. 2012, 3485–3491. 10.1002/ejoc.201200256.

[ref31] Reisi-Vanani A.; Rezaei A. A. Evaluation of the aromaticity of non-planar and bowl-shaped molecules by NICS criterion. J. Mol. Graphics Modell. 2015, 61, 85–88. 10.1016/j.jmgm.2015.05.015. 26188797

[ref32] Solà M. Connecting and combining rules of aromaticity. Towards a unified theory of aromaticity. Wiley Interdiscip. Rev.: Comput. Mol. Sci. 2019, 9, e1404 10.1002/wcms.1404.

[ref33] Schleyer P. V. R.; Maerker C.; Dransfeld A.; Jiao H.; van Eikema Hommes N. J. R. Nucleus-Independent Chemical Shifts: A Simple and Efficient Aromaticity Probe. J. Am. Chem. Soc. 1996, 118, 6317–6318. 10.1021/ja960582d. 28872872

[ref34] Geuenich D.; Hess K.; Köhler F.; Herges R. Anisotropy of the Induced Current Density (ACID), a General Method To Quantify and Visualize Electronic Delocalization. Chem. Rev. 2005, 105, 3758–3772. 10.1021/cr0300901. 16218566

[ref35] Hückel E. Quantentheoretische Beiträge zum Benzolproblem I. Die Elektronenkonfiguration des Benzols und verwandter Verbindungen. Z. Phys. 1931, 70, 204–286. 10.1007/bf01339530.

[ref36] Hückel E. Quanstentheoretische Beiträge zum Benzolproblem II. Quantentheorie der induzierten Polaritäten. Z. Phys. 1931, 72, 310–337. 10.1007/bf01341953.

[ref37] Breslow R. Antiaromaticity. Acc. Chem. Res. 1973, 6, 393–398. 10.1021/ar50072a001.

[ref38] Kollmar H.; Staemmler V. Theoretical Study of the Structure of Cyclobutadiene. J. Am. Chem. Soc. 1977, 99, 3583–3587. 10.1021/ja00453a009.

[ref39] Bally T. Cyclobutadiene: The Antiaromatic Paradigm?. Angew. Chem., Int. Ed. 2006, 45, 6616–6619. 10.1002/anie.200602279. 16986189

[ref40] Zdetsis A. D. 4 n + 2 = 6 n ? A Geometrical Approach to Aromaticity?. J. Phys. Chem. A 2021, 125, 6064–6074. 10.1021/acs.jpca.1c02872. 34232038

[ref41] Zdetsis A. D. Classics Illustrated: Clar’s Sextet and Hückel’s $(4n + 2)$ π-Electron Rules. J. Phys. Chem. C 2018, 122, 17526–17536. 10.1021/acs.jpcc.8b05020.

[ref42] Hedberg L.; Hedberg K.; Cheng P.-c.; Scott L. T. Gas-Phase Molecular Structure of Corannulene, C 20 H 10 . An Electron-Diffraction Study Augmented by ab Initio and Normal Coordinate Calculations. J. Phys. Chem. A 2000, 104, 7689–7694. 10.1021/jp0015527.

[ref43] Hirsch A.; Chen Z.; Jiao H. Spherical Aromaticity inIh Symmetrical Fullerenes: The 2(N+1)2 Rule. Angew. Chem., Int. Ed. 2000, 39, 3915–3917. 10.1002/1521-3773(20001103)39:21<3915::aid-anie3915>3.0.co;2-o. 29711706

[ref44] Poater J.; Solà M. Open-shell spherical aromaticity: the 2N2 + 2N + 1 (with S = N + 1.2) rule. Chem. Commun. 2011, 47, 11647 10.1039/c1cc14958j. 21952479

[ref45] Popov I. A.; Bozhenko K. V.; Boldyrev A. I. Is graphene aromatic?. Nano Res. 2012, 5, 117–123. 10.1007/s12274-011-0192-z.

[ref46] Kumar A.; Duran M.; Solà M. Is coronene better described by Clar’s aromatic π-sextet model or by the AdNDP representation?. J. Comput. Chem. 2017, 38, 1606–1611. 10.1002/jcc.24801. 28394019

[ref47] Fedik N.; Boldyrev A. I. Insight into The Nature of Rim Bonds in Coronene. J. Phys. Chem. A 2018, 122, 8585–8590. 10.1021/acs.jpca.8b07937. 30296096

[ref48] Roos B. O.; Taylor P. R.; Sigbahn P. E. M. A complete active space SCF method (CASSCF) using a density matrix formulated super-CI approach. Chem. Phys. 1980, 48, 157–173. 10.1016/0301-0104(80)80045-0.

[ref49] Werner H. J.; Meyer W. A quadratically convergent multiconfiguration–self-consistent field method with simultaneous optimization of orbitals and CI coefficients. J. Chem. Phys. 1980, 73, 2342–2356. 10.1063/1.440384.

[ref50] Hehre W. J.; Ditchfield R.; Pople J. A. Self-Consistent Molecular Orbital Methods. XII. Further Extensions of Gaussian-Type Basis Sets for Use in Molecular Orbital Studies of Organic Molecules. J. Chem. Phys. 1972, 56, 2257–2261. 10.1063/1.1677527.

[ref51] Lee C.; Yang W.; Parr R. G. Development of the Colle-Salvetti correlation-energy formula into a functional of the electron density. Phys. Rev. B: Condens. Matter Mater. Phys. 1988, 37, 785–789. 10.1103/physrevb.37.785. 9944570

[ref52] Becke A. D. Density-functional exchange-energy approximation with correct asymptotic behavior. Phys. Rev. A 1988, 38, 3098–3100. 10.1103/physreva.38.3098. 9900728

[ref53] Becke A. D. Density-functional thermochemistry. III. The role of exact exchange. J. Chem. Phys. 1993, 98, 5648–5652. 10.1063/1.464913.

[ref54] Frisch M. J.; Trucks G. W.; Schlegel H. B.; Scuseria G. E.; Robb M. A.; Cheeseman J. R.; Scalmani G.; Barone V.; Petersson G. A.; Nakatsuji H.; Gaussian 16, Revision B.01, 2016.

[ref55] Weigend F.; Ahlrichs R. Balanced basis sets of split valence, triple zeta valence and quadruple zeta valence quality for H to Rn: Design and assessment of accuracy. Phys. Chem. Chem. Phys. 2005, 7, 3297–3305. 10.1039/b508541a. 16240044

[ref56] Weigend F. Hartree–Fock exchange fitting basis sets for H to Rn. J. Comput. Chem. 2008, 29, 167–175. 10.1002/jcc.20702. 17568435

[ref57] Neese F. Software update: the ORCA program system, version 4.0. Wiley Interdiscip. Rev.: Comput. Mol. Sci. 2018, 8, e1327 10.1002/wcms.1327.

