# Supplementary material for: Toward a Generalized Hückel Rule: The Electronic Structure of Carbon Nanocones
Source: J Phys Chem A. 2021 Nov 4;125(45):9819–25. doi: 10.1021/acs.jpca.1c06402 (PMC8607423; doi:10.1021/acs.jpca.1c06402)
Supplement: Supplementary file 2 — jp1c06402_si_002.pdf [file jp1c06402_si_002.pdf]

Supporting Information for: Towards a Generalized Hückel Rule: The Electronic Structure of Carbon Nanocones

Yusuf Bramastya Apriliyanto,[†,‡] Stefano Battaglia,[*,¶] Stefano Evangelisti,[*,†] Noelia Faginas-Lago,[‡] Thierry Leininger,[†] and Andrea Lombardi[‡]

† *Laboratoire de Chimie et Physique Quantiques - IRSAMC, Université de Toulouse et CNRS, 118, Route de Narbonne, F-31062 Toulouse Cedex - France*

‡ *Dipartimento di Chimica, Biologia e Biotecnologie, Università degli Studi di Perugia, Via Elce di Sotto 8, I-06123 Perugia - Italy*

¶ *Department of Chemistry-BMC, Uppsala University, P.O. Box 576, SE-75123 Uppsala - Sweden*

E-mail: stefano.battaglia@kemi.uu.se; stefano.evangelisti@univ-tlse3.fr

Additional Computational Details

In this Section, the computational details concerning the tight-binding approach are discussed.

Tight-binding

All the computations at the tight-binding level (Hückel calculations in the language of chemists) have been performed by using a self-developed Fortran code. The Hückel Hamiltonian has been constructed by starting from the connectivity of every carbon atom in each concentric ring and then connecting the different rings together. As usual, the elements are defined as

$$\langle i|\hat{H}|i\rangle = \alpha = 0 \tag{1}$$

and

$$\langle i|\hat{H}|j\rangle = \beta\gamma_{ij} \tag{2}$$

Here γ_{ij} is equal to 1 if i and j are different sites that are connected in the nanostructure skeleton, and 0 otherwise. Notice that the hydrogens are not considered at the tight-binding level. Since the C–C bonds have very similar length in all our structures, we assumed the same value of β for all the topologically connected pairs of atoms. For this reason, and without loss of generality, in all our calculations the parameter β was fixed at the value $\beta = -1$. We remind that $-\beta$ is sometimes called the hopping integral in the physics literature, and it is usually indicated as t.

For some systems, a set of m strictly zero eigenvalues at the Fermi level has been obtained after diagonalization of the Hamiltonian. Because of the alternating symmetry, and the fact that all the systems considered in the present work have zero total charge, the m zero-energy orbitals host a total of m electrons. In that case we considered the system as an open shell one, and each associated eigenvector was considered as a half-filled orbital.

Ab Initio

The geometries of the optimized structures are available as xyz files.
